# Investigating a severe acute malnutrition outbreak in Dubti District, Awsiresu Zone, Afar Region, Northeast Ethiopia (2022)

**DOI:** 10.3389/fpubh.2024.1475104

**Published:** 2024-11-07

**Authors:** Abiyie Demelash Gashe, Dawit Zenebe Woldemichael, Fentahun Agegnehu Worku, Kedir Ali Mahmud, Aman Yesuf Endries

**Affiliations:** ^1^Ethiopian Field Epidemiology Training Program, St. Paul’s Hospital Millennium Medical College, Addis Ababa, Ethiopia; ^2^Department of Epidemiology, Mekelle University, Mekelle, Ethiopia; ^3^Afar Public Health Institute, Samara, Ethiopia; ^4^Department of Epidemiology, St. Paul’s Hospital Millennium Medical College, Addis Ababa, Ethiopia

**Keywords:** malnutrition, severe acute malnutrition, outbreak, investigation, risk factors, Ethiopia

## Abstract

**Introduction:**

Ethiopia is a global hotspot for child malnutrition, with an estimated 1.2 million children under five affected by severe acute malnutrition (SAM) in 2022. In response, the country has integrated SAM into its broader disease surveillance system. In January 2022, the Dubti District Health Office in the Afar Region detected an unusual surge in SAM cases through its surveillance system. This study aimed to assess the extent of the outbreak and identify the associated risk factors.

**Methods:**

We conducted an unmatched case–control study involving 258 mother–child dyads from five affected kebeles in the Dubti District of the Afar Region Ethiopia. The descriptive study included all 442 SAM cases from the line list, while 86 cases and 168 controls were selected using a simple random sampling method for the analytic study. The data were entered into EpiData software (version 3.1) and analyzed using SPSS software (version 25.0). Binary logistic regression (LR) analysis was performed to identify risk factors for SAM. Statistically, the results were summarized using an adjusted odds ratio (AOR), 95% confidence intervals (CIs), and a *p*-value of <0.05.

**Results:**

The median age of the cases was 22 months, with an interquartile range of 12–34 months. A total of 39 deaths were reported, with a case fatality rate (CFR) of 8.82%. The identified SAM risk factors included households with more than five members (AOR = 3.341, 95% CI: 1.475–7.563), more than five under-five children (AOR = 4.442, 95% CI: 2.000–9.866), lack of vaccination (AOR = 3.641, 95% CI: 1.618–8.198), pneumonia (AOR = 5.61, 95% CI: 2.488–12.651), diarrhea (AOR = 4.68, 95% CI: 2.169–10.097), lack of access to sanitation and hygiene (AOR = 3.18, 95% CI: 1.462–6.934), and household food insecurity (AOR = 9.46, 95% CI: 2.095–42.712).

**Conclusion:**

The study revealed a significant outbreak of SAM, with a CFR of 8.82%. The outbreak was associated with factors such as large family sizes, having multiple under-five children, a lack of vaccination, pneumonia, and diarrhea. These findings emphasize the urgent need to safeguard essential child health services, water supply, sanitation and hygiene, and household food security.

## Introduction

Child undernutrition includes various nutritional disorders, such as underweight, wasting, stunting, and micronutrient deficiencies (1). It is a critical issue for child survival and significantly impacts both cognitive and physical development (2, 3). Undernutrition can manifest in acute, chronic, or mixed forms. Severe acute malnutrition (SAM), characterized by severe wasting and recent weight loss, is a severe form of protein-energy deficiency (1, 4).

The causes of malnutrition can be categorized into three types of causes, namely immediate, underlying, and basic (5). Basic causes of malnutrition are rooted in the political, social, and economic environment. Underlying causes include insufficient food access, inadequate maternal and child care, and poor water, sanitation, and hygiene (WASH) conditions. Immediate causes include inadequate dietary intake and acute illnesses (5–7).

Malnutrition contributes to 50% of all child deaths. Malnourished children face an increased risk of hospitalization and contracting infectious diseases, such as diarrhea, acute respiratory infections, measles, and malaria (8).

Globally, in 2022, it was reported that 45 million under-five children were wasted, with only 7.3 million receiving treatment for SAM, and 149 million were stunted (9, 10). According to the United Nations International Children’s Emergency Fund (UNICEF), nearly 40 million under-five children are at risk of SAM, with approximately one child developing SAM every minute in 15 crisis-hit countries, including Afghanistan, Haiti, Yemen, Burkina Faso, Chad, the Democratic Republic of the Congo, Kenya, Madagascar, Mali, Niger, Nigeria, Somalia, South Sudan, Sudan, and Ethiopia (11).

The World Health Organization (WHO) reported that 11 million under-five children were acutely malnourished during 2022, specifically in seven countries of the Greater Horn of Africa, including Djibouti, Somalia, Sudan, South Sudan, Ethiopia, Kenya, and Uganda. The WHO also reported that the SAM rates were 5–24% in Somalia, 2.7% in Sudan, 1.3–6.1% in South Sudan, 2–12.3% in Kenya, 0.9–4.5% in Uganda, and 11.1–14.7% in Djibouti (12).

In Ethiopia, a UNICEF report indicates that approximately 4.7 million under-five children are malnourished, including 1.2 million children with SAM. In addition, 5.5 million children are stunted, with 1.8 million experiencing severe stunting. Undernutrition accounts for 45% of all child deaths in the country (13).

Ethiopia has pledged to end child malnutrition by 2030 through initiatives such as integrating nutritional surveillance into the national Integrated Diseases Surveillance and Response system, incorporating targets into the National Health Sector Transformation Plan, and adopting the United Nations’ Sustainable Development Goal 2 (14, 15). As a result, significant progress has been made in reducing wasting, stunting, and underweight (16). The prevalence rates of stunting, severe wasting, and underweight in the country are 37, 7, and 21%, respectively (17). However, recent conflicts, droughts, and environmental changes have exacerbated nutritional problems, particularly in three conflict-affected regions of Ethiopia: Tigray, Amhara, and Afar (18).

SAM is a weekly reportable condition under public health emergency management (PHEM). In January 2022, the Dubti District Health Office in the Afar Region noted an unusual surge in SAM cases through its routine surveillance system, following the reporting of 17 SAM-related deaths. A multidisciplinary team, comprising field epidemiologists, health officers, PHEM officers, and health information technicians, was subsequently deployed. Data were analyzed, an action threshold level was established, and an outbreak was confirmed, leading to a prompt response.

## Materials and methods

### Study setting

An unmatched case–control study was conducted in five SAM-affected kebeles (such as Korile, Dembel, Gumtameli, Sekoyta, and Galimeda), which are small administrative units in Dubti District of Afar, Northeastern Ethiopia, from 1 May 2022 to 30 May 2022. This area is located 600 km from Addis Ababa, the capital city of Ethiopia. The district has 13 kebeles and 10,992 households. Based on 2007 census data, the total population of the district for 2022–2023 was estimated to be 49,173. The district is one of the hotspot areas for under-five malnutrition in the region, characterized by recurrent droughts and pastoral communities that rely on livestock production. The prevalence rates of wasting, stunting, and underweight were estimated to be 16.2, 43.1, and 24.8%, respectively (19).

### Study population

All under-five children living in the five malnutrition-affected kebeles in Dubti District comprised the study population. Cases were defined as children aged 6–59 months with a weight-for-height score (WFH) of less than −3 standard deviations (SDs), a mid-upper arm circumference (MUAC) of less than 110 mm, or bilateral pitting edema (20). Controls were defined as children of the same age with WFH score greater than −2 SDs and/or MUAC greater than 125 mm (21). Children with congenital anomalies, including Down syndrome, and physical deformities that interfered with the standard anthropometric procedure, as well as those whose mothers or caregivers failed to provide informed consent, were excluded from the study.

### Sample size determination

We used all SAM cases identified in the line list for descriptive analysis. For the analytic study, the sample size was calculated using Epi-Info software version 7.1.1.0 based on the following assumptions: power (80%), 95% confidence interval (CI), a case-to-control ratio of 1:2, and findings from a previous study that identified prelacteal feedings as risk factors for SAM (19). Therefore, by considering the percentage of controls exposed (78.6%), the percentage of cases exposed (93.3%), an odds ratio of 3.81, and a 10% non-response rate, the final calculated sample size was 258, comprising 86 cases and 172 controls.

Mathematically, $N=\frac{r+1\left(p-q-\right)\left(Z\beta +\frac{Z\alpha /2}{2}\right)}{r\left(p1-p2\right)}+10\%$ non-response rate.

where *N* = sample size, P1 = percentage of cases exposed (93.3%), P2 = percentage of controls exposed (78.6%), the odds ratio (OR) = 3.81, r = ratio of cases to controls (1:2), Zβ = 80%, Zα/2 = 1.96, p^−^ = (P1 + r × P2)/(r + 1), and q^−^ = 1–p^−^.

### Sampling procedure

All five affected kebeles—Korile, Dembel, Gumtameli, Sekoyta, and Galimeda—were purposefully selected for the investigation. To describe the SAM outbreak by person, place, and time, we utilized the entire line list, which included all SAM reports submitted to the Dubti District Health Office during the outbreak period. However, when investigating the factors associated with the SAM outbreak, all SAM cases in the line list were identified and assigned unique identification numbers. SAM-affected children in these kebeles were then selected using a simple random sampling technique from the line list. These children were then traced back to their communities for data collection. Controls—children who did not meet the standard case definition of SAM—were also recruited using a simple random sampling technique from neighbors living in the same residential areas. For every SAM-affected child, two neighbor controls were recruited.

### Confirmation of the outbreak

A SAM outbreak occurs when the number of SAM cases exceed the threshold during a normal season in a specific area. The WHO recommends various threshold calculation techniques for weekly reportable diseases, such as the 75th percentile, mean + 2 SDs, cumulative sum, and a constant case count (22). Considering that Dubti District is an area endemic for child undernutrition, we used the mean + 2 SD method, which adds 2 SDs to the average number of reported SAM cases over the past 5 years. Using District Health Information Software, the current data (2021/2022) were compared to the average weekly SAM reports from 2017 to 2021 to determine whether the action threshold was surpassed.

### Data collection procedure and measurement

We used a structured questionnaire adapted from the literature (19, 21, 23–27) and conducted a house-to-house survey to collect data from mothers/caregivers through face-to-face interviews, immunization cards, and anthropometric measurements. The child’s age was estimated using an immunization card, a birth certificate, or information recalled by the mothers or caregivers. Dietary diversity was assessed through 24-h food recall of seven WHO-recommended food items.

Dietary diversity was assessed using the dietary diversity score (DDS) based on 24-h food recall, in accordance with the WHO’s minimum dietary diversity recommendations. A child was considered to have a diversified diet if they consumed four or more food items from the following seven WHO-recommended food groups: (1) grains, roots, and tubers; (2) legumes and nuts; (3) dairy products, such as milk, yogurt, and cheese; (4) flesh foods, including meat, fish, poultry, and liver/organ meats; (5) eggs; (6) vitamin A-rich fruits and vegetables; and (7) other fruits and vegetables. A DDS of ≥4 was considered indicative of a diversified diet (26).

Household food security was measured using the Household Food Insecurity Access Scale (HFIAS). The HFIAS consists of two types of related questions: nine occurrence questions that ask about experiences of food insecurity in the past 4 weeks (30 days) and 9 severity questions that inquire about the frequency of these experiences. Furthermore, the HFIAS categorizes household food insecurity into four categories: category one, food security if [(Q1a = 0 or Q1a = 1) and Q2 = 0 and Q3 = 0 and Q4 = 0 and Q5 = 0 and Q6 = 0 and Q7 = 0 and Q8 = 0 and Q9 = 0]; category two, mildly food insecure access if [(Q1a = 2 or Q1a = 3 or Q2a = 1 or Q2a = 2 or Q2a = 3 or Q3a = 1 or Q4a = 1) and Q5 = 0 and Q6 = 0 and Q7 = 0 and Q8 = 0 and Q9 = 0]; category three, moderately food insecure access if [(Q3a = 2 or Q3a = 3 or Q4a = 2 or Q4a = 3 or Q5a = 1 or Q5a = 2 or Q6a = 1 or Q6a = 2) and Q7 = 0 and Q8 = 0 and Q9 = 0]; and category four, severely food insecure access if [Q5a = 3 or Q6a = 3 or Q7a = 1 or Q7a = 2 or Q7a = 3 or Q8a = 1 or Q8a = 2 or Q8a = 3 or Q9a = 1 or Q9a = 2 or Q9a = 3], as described in detail in the HFIAS (24). Households in category 1, with an HFIAS score of 0–1, were considered food secure, whereas those in categories two, three, or four were classified as food insecure (27).

The child’s vaccination status was assessed using an immunization card and information recalled by the mothers. A child who received all of the vaccines recommended for their age was considered fully immunized (28). A child who presented with a cough, fast breathing, and/or danger signs, based on the integrated management of newborn and child illness classification, was diagnosed with pneumonia (29). Furthermore, diarrhea was defined as passing three or more loose or liquid stools per day (30). Five nurses with a Bachelor of Science degree who had experience in under-five nutritional surveys and two supervisors with a master’s degree in Public Health participated in the data collection.

### Anthropometric measurement

The control children underwent standardized anthropometric measurements. Briefly, weight was recorded using a calibrated portable scale to the nearest 0.1 kg, with participants wearing light clothing. Height was measured with a calibrated portable stadiometer to the nearest 0.1 cm. The participants stood without shoes, in a Frankfurt position, with their heels, buttocks, shoulders, and heads touching a vertical support. For the children aged 6–23 months, recumbent length was measured. The mid-upper arm circumference (MUAC) was determined by measuring the circumference of the upper arm at its midpoint, with the arm bent at a right angle (20).

### Data quality control

We used the English version of the questionnaire, which was translated into the local language, Afarigna, and then back into English. The questionnaire was pretested with 5% of the sample to ensure clarity, completeness, and consistency. Anthropometric indices were measured and interpreted according to the WHO 2006 growth standards (20). The data collectors received 3 days of training on the data collection tool and procedure, as well as on protecting data confidentiality.

### Data analysis

The data were entered into EpiData software version 3.1 and analyzed using SPSS software version 25.0. A chi-squared test was conducted to assess differences in the baseline sociodemographic and economic characteristics of the cases and controls. However, when the conditions for the chi-squared test were violated—specifically, when the expected values in at least 80% of the cells were less than five or when any cell had an expected value less than one—Fisher’s exact test was conducted. A binary logistic regression (LR) analysis model was applied to identify risk factors for SAM. Variables with corresponding *p*-values <0.25 in the bivariable binary LR analysis were further analyzed. Adjusted odds ratios (AORs) with 95% confidence intervals (CIs) were calculated, and a *p*-value of <0.05 was considered statistically significant. The overall model fit was assessed using the Hosmer–Lemeshow goodness-of-fit test, with a p-value of >0.05. In addition, the data were assessed for collinearity with a variance inflation factor of less than 5.

### Ethical consideration

Ethical clearance was obtained from the Afar Public Health Institute, ethical approval number APH015/2022. Informed consent was obtained from all individual participants included in the study. The data confidentiality was assured via the de-identification of personal identifier information and the storage of the file in a secure folder. Children who met the case definition of SAM during control selection were linked to nearby health facilities for nutritional intervention.

## Results

### Descriptive epidemiology

#### Description of the SAM cases by person

A total of 442 SAM cases were reported in this outbreak. Of these, 245 (55.4%) were male patients. The median age of the cases was 22 months, with an interquartile range of 12–34 months. A total of 191 (43.2%) children presented with diarrhea, 138 (31.2%) with fever, and 185 (41.9%) with pneumonia.

The incidence of SAM was 284.6 per 1,000 population. The male patients had the highest attack rate (AR; 320/1,000 population), followed by those aged 6–11 months (330/1,000 population). Furthermore, 39 SAM-related deaths were recorded, resulting in a case fatality rate (CFR) of 8.82%. The highest CFR was observed among the female patients (10.6%), followed by those aged 6–11 months (10.5%; Table 1).

**Table 1:** SAM AR and CFR in Dubti District, Awsiresu Zone, Afar region, Northeastern Ethiopia, 2022.

Variables	Classification	Total under-five population (*N* = 1,553)	SAM cases (*N* = 442)	Deaths (*N* = 39)	AR/1,000 population	CFR (%)
Sex	Male	765	245	18	320/1,000	7.3%
	Female	788	197	21	250/1,000	10.6%
Age	6–11 months	318	105	11	330/1,000	10.5%
	12–36 months	807	215	18	266/1,000	8.4%
	37–59 months	428	122	10	285/1,000	8.2%
Total population		1,553	442	39	284.6/1,000	8.82%

### Description of the SAM cases by place

A total of 260 (58.9%) cases were reported from Galimeda, followed by Korile with 59 cases (13.3%) and Debel with 45 cases (10.2%). Similarly, the highest AR was observed in Galimeda (350/1,000 population), followed by Korile (309/1,000 population) and Debel (300/1,000 population; Table 2).

**Table 2:** SAM rates by affected Kebeles in Dubti District, Awsiresu Zone, Afar, Northeastern Ethiopia, 2022.

Place (Kebeles)	Cases (*N* = 442)		Total under-five population	AR/1,000 population
	Frequency	Percent		
Korile	59	13.3%	191	309/1,000
Debel	45	10.2%	150	300/1,000
Gumtameli	43	9.7%	259	166/1,000
Sekoyta	35	7.9%	211	166/1,000
Galimeda	260	58.9%	742	350/1,000

### Description of the SAM outbreak by time

**Figure 1:**
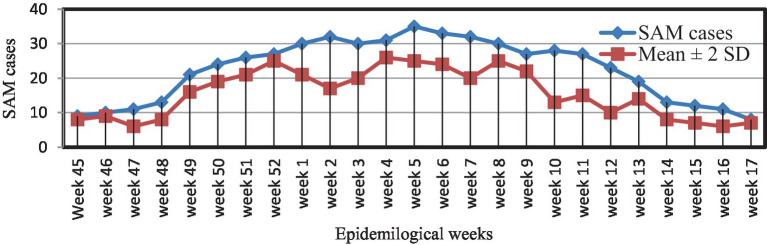
Weekly SAM reports and action thresholds in Dubti District, Awsiresu Zone, Afar Region, Northeastern Ethiopia, 2022.

Using the mean + 2 SDs method, it was found that the threshold level was surpassed from epidemiological week (Epi-week) 45 in 2021 to Epi-week 17 in 2022, confirming the SAM outbreak (Figure 1).

**Figure 2:**
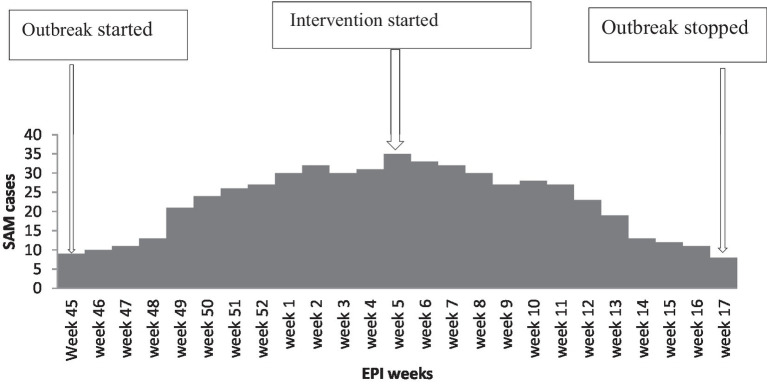
Epidemic curve depicts the onset of SAM in Dubti District, Awsiresu Zone, Afar Region, Northeastern Ethiopia, 2022.

The SAM outbreak began in Epi-week 45 in 2021 and continued through Epi-week 5 in 2022, when it dropped below the action threshold level in Epi-week 17 in 2022. The epidemic curve suggested a continuous common-source type of outbreak (Figure 2).

### Analytic study

#### Sociodemographic characteristics

The study had 258 mother–child pairs (86 cases and 172 controls), with 84 cases and 168 controls willing to participate. The response rate was 97.7%. The median ages of the cases and controls were 24 and 26 months, respectively. There was a statistically significant difference in educational level, occupation, family size, and the number of children under 5 years of age between cases and controls at a *p*-value of <0.005 (Table 3).

**Table 3:** Sociodemographic characteristics of the mothers/caregivers in Dubti District, Awsiresu Zone, Afar Region, Northeast Ethiopia, 2022.

Variables	Cases (*N* = 84)	Controls (*N* = 168)	Chi-squared (X^2^)	
	Frequency (percent)	Frequency (percent)	X^2^	P-value
Sex of the child			0.071	0.789
Male	43 (51.2%)	83 (49.4%)		
Female	41 (48.8%)	85 (50.6%)		
Age of the mothers			0.1202	0.749
< 18 years	9 (10.7%)	24 (14.3%)		
18–24 years	18 (21.4%)	36 (21.4%)		
25–34 years	32 (38.1%)	59 (35.1%)		
≥ 35 years	25 (29.8%)	49 (29.2%)		
Marital status			0.69	0.876
Married	73 (86.9%)	145 (86.4%)		
Divorced	6 (7.1%)	12 (7.1%)		
Widowed	5 (6%)	11 (6.5%)		
Ethnicity			0.034	0.983
Afar	64 (76.2%)	125 (74.4%)		
Amhara	8 (9.5%)	19 (11.3%)		
Tigre	12 (14.3%)	24 (14.3%)		
Religion			2.034	0.362***
Muslim	70 (83.3%)	143 (85.1%)		
Orthodox	13 (15.5)	25 (14.9%)		
Protestant	1 (1.2%)	0 (0%)		
Education			71.09	< 0.001
No formal education	59 (70.2%)	102 (60.7%)		
Primary (1–8)	17 (20.2%)	44 (26.3%)		
Secondary (9–12)	4 (4.8%)	14 (8.3%)		
College and above	4 (4.8%)	8 (4.7%)		
Occupation			7.569	0.023
Housewife	42 (50%)	85 (50.6%)		
Herd livestock	37 (44%)	73(43.5%)		
Employed	5 (6%)	10(5.9%)		
Family size			22.36	< 0.001
≤ 2	10 (11.9%)	29 (17.3%)		
3−4	17(20.2%)	50(29.8%)		
>5	57 (67.9%)	89 (52.9%)		
Number of under-five children			26.11	< 0.001
≤ 2	28 (33.3%)	48 (28.6%)		
3−4	40 (47.7%)	50 (29.8%)		
>5	16 (19%)	89 (53%)		

***Fisher’s exact test.

#### Household food security status

According to the HFIAS, four (4.8%) of the households among the cases and 27 (16.1%)of the households among the controls were food secure, whereas 80 (95.2%) households among the cases and 141 (83.9%) households among the controls were food insecure. Among the food-insecure households in the case group, 37 (44%) were classified as mildly food insecure, 25 (29.8%) as moderately food insecure, and 18 (21.4%) as severely food insecure. Furthermore, 70 (41.7%), 51 (30.3%), and 20 (11.9%) of the food-insecure households in the control group were classified as mildly insecure, moderately insecure, and severely food insecure, respectively (Table 4).

**Table 4:** Household food security status in Dubti District, Awsiresu Zone, Afar Region, Northeast Ethiopia, 2022.

Questions	Cases (*N* = 84)				Controls (*N* = 168)			
	Yes			No	Yes			No
	Rarely(1)	Sometimes(2)	Often(3)		Rarely(1)	Sometimes(2)	Often(3)	
HH member worries about not having enough food	4(4.8%)	36(42.8%)	40(47.6%)	4(4.8%)	27(16%)	68(40.5%)	46(27.5%)	27(16%)
HH member not able to eat preferred foods	9(10.7%)	34(40.5%)	31(36.9%)	10(36.9%)	51(30.3%)	50(29.8%)	30(17.9%)	37(22%)
HH member eats a limited variety of foods	13(15.5%)	35(41.6%)	23(27.4%)	13(15.5%)	47(27.9%)	50(29.8%)	38(22.7%)	33(19.6%)
HH member eats foods they do not want to eat	19(22.6%)	26(31%)	31(36.9%)	8(9.5%)	62(36.9%)	38(22.6%)	32(19.1%)	36(21.4%)
HH member eats a smaller meal than needed	14(16.7%)	18(21.4%)	40(47.6%)	12(14.3%)	69(41%)	48(28.6%)	23(13.7%)	28(16.7%)
HH member eats fewer meals in a day	14(16.7%)	30(35.7%)	29(34.5%)	11(13.1%)	55(32.7%)	43(25.6%)	41(24.4%)	29(17.3%)
There was never any food to eat of any kind in the HH	32(38%)	25(29.7%)	22(26.2%)	5(5.9%)	57(33.9%)	33(19.6%)	40(23.8%)	38(22.7%)
HH member goes to sleep at night hungry	24(28.6%)	31(36.9%)	23(27.4%)	6(7.1%)	68(40.5%)	38(22.6%)	34(20.2%)	28(16.7%)
HH member goes day and night without eating	37(44%)	16(19%)	14(16.7%)	17(20.3%)	61(36.3%)	39(23.2%)	27(16.1%)	41(24.4%)
HFIAS category								
Food secure	4 (4.8%)				27 (16.1%)			
Food insecure	80 (95.2%)				141 (83.9%)			
Mildly insecure	37(44%)				70 (41.7%)			
Moderately insecure	25(29.8%)				51 (30.3%)			
Severely insecure	18(21.4%)				20 (11.9%)			

#### Child dietary diversity practices

According to the WHO’s minimum dietary diversity recommendation, 32 (38%) of the cases and 92 (54.8%) of the controls met these standards. Grains, roots, and tubers were the most commonly consumed foods, with 546 (4.3%) of the cases and 140 (76.2%) controls consuming them. This was followed by legumes and nuts, with 46 (54.8%) of the cases and 135 (65.5%) of the controls consuming them. Among the severely malnourished children, 20 (23.8%), 26 (31%), and 23 (27.4%) consumed eggs, flesh foods, and vitamin A-rich fruits and vegetables, respectively (Table 5).

**Table 5:** Dietary diversity practices among the children in the Dubti district, Awsiresu Zone, Afar Region, Northeast Ethiopia, 2022.

Items (child’s diet)	Responses	Cases (*N* = 84)		Controls (*N* = 168)	
		Frequency	Percent	Frequency	Percent
Grains, roots, and tubers	Yes	54	64.3%	140	76.2%
	No	30	35.7%	28	23.8%
Legumes and nuts	Yes	46	54.8%	135	65.5%
	No	38	45.2%	33	34.5%
Dairy products	Yes	14	16.7%	58	40.5%
	No	70	83.3%	110	59.5%
Flesh foods	Yes	26	31%	64	38.1%
	No	58	69%	104	61.9%
Eggs	Yes	20	23.8%	70	58.3%
	No	64	76.2%	98	41.7%
Vitamin A-rich fruits and vegetables	Yes	23	27.4%	62	48.8%
	No	61	72.6%	106	51.2%
Other fruits and vegetables	Yes	25	29.8%	43	25.6%
	No	59	70.2%	145	74.4%
DDS	< 4	52	62%	76	45.2%
	≥ 4	32	38%	92	54.8%

### Child feeding practices

Breastfeeding was initiated within 1 h of delivery for 67 (79.8%) of the cases and 138 (82.1%) of the controls. During the first 6 months of life, 55 (65.8%) of the cases and 108 (64.3%) of the controls were fed only breast milk. In addition, 33 (35.7%) of the cases and 111 (66%) of the controls received more than four feeds per day (Table 6).

**Table 6:** Household access to WASH, child feeding practices, and health-related characteristics in Dubti District, Awsiresu Zone, Afar Region, Northeast Ethiopia, 2022.

Variables	Cases (*N* = 84)	Controls (*N* = 168)
	Frequency (percent)	Frequency (percent)
Birth order		
First	15 (17.9%)	35 (20.8%)
Second to fourth	19 (22.6%)	29 (17.2%)
Fifth and above	50 (59.5%)	104 (61.9%)
Breastfeeding initiation		
Within 1 h	67 (79.8%)	138 (82.1%)
Hours later	17 (20.2%)	30 (17.9%)
Prelacteal feeding		
Yes	19 (22.6%)	38 (22.6%)
No	65 (77.4%)	130 (77.4%)
Colostrum feeding		
Yes	67 (79.8%)	126 (75%)
No	17 (20.2%)	42 (25%)
Exclusive breastfeeding during the first 6 months		
Yes	55 (65.8%)	108 (64.3%)
No	29 (34.5%)	60 (35.7%)
Complimentary feeding during the first 6 month		
Yes	18 (21.4%)	65 (38.7%)
No	66 (78.6%)	103 (61.3%)
Child feeding		
< 4 times/day	54 (64.3%)	57 (34%)
≥ 4 times/day	30 (35.7%)	111 (66%)
Child Immunization		
Completed vaccination	19 (22.6%)	93 (55.4%)
Partially vaccinated or not vaccinated at all	65 (77.4%)	75 (44.6%)
Pneumonia		
Yes	54 (64.3%)	53 (31.5%)
No	30 (35.7%)	115 (68.5%)
Diarrheal disease		
Yes	60 (71.4%)	53 (31.5%)
No	24 (28.6%)	115 (68.5%)
Fever		
Yes	12 (14.3%)	13 (7.7%)
No	72 (85.7%)	155 (92.3%)
Access to drinking water		
Yes	34 (40.5%)	84 (50%)
No	50 (59.5%)	84 (50%)
Access to sanitation and hygiene		
Yes	37 (45%)	119 (70.8%)
No	47 (56%)	49 (29.2%)
Presence of a latrine		
Yes	32 (38%)	100 (59.5%)
No	52 (69%)	68 (40.5%)

### Child immunization and medical illness

A total of 19 (22.6%) of the cases and 93(55.4%) of the controls were fully immunized for their age. Furthermore, 60 (71.4%) of the cases and 53 (31.5%) of the controls had diarrhea, while 54 (64.3%) of the cases and 53 (31.5%) of the controls had acquired pneumonia (Table 6).

### Household access to WASH

A total of 34 (40.5%) of the households in the case group and 84 (50%) of the households in the control group reported having access to safe drinking water. Similarly, 37 (45%) of the households in the case group and 119 (70.8%) of the households in the control group reported access to sanitation and hygiene (Table 6).

### Factors associated with the SAM outbreak

After controlling for potential confounding factors, the children in families with more than five members had 3.34 times greater odds of experiencing SAM compared to the children in smaller families (AOR = 3.34, 95% CI: 1.475−7.563). Similarly, households with more than five under-five children had 4.4 times greater odds of SAM than their counterparts (AOR = 4.44, 95% CI: 2.000−9.866). Compared to the fully vaccinated children, unvaccinated children were 3.6 times more likely to experience SAM (AOR = 3.64, 95% CI: 1.618 ~ 8.198). The children with a history of pneumonia had a 5.6-fold greater risk of experiencing SAM (AOR = 5.61, 95% CI: 2.488−12.651), while those with diarrheal disease had a 4.7-fold greater chance of experiencing SAM (AOR = 4.68, 95% CI: 2.169−10.097; Table 7).

**Table 7:** Factors associated with the SAM outbreak in Dubti District, Awsiresu Zone, Afar Region, Northeast Ethiopia, 2022.

Variables	Bivariable binary LR		Multivariable binary LR	
	COR	*p*-value	AOR (95% CI)	*p*-value
Occupation				
Housewife				
Herd livestock	3.30	0.028	6.804 (0.451–31.916)	0.15
Employed	1		1	
Family size				
≤ 5	1		1	
> 5	3.85	< 0.001	3.341 (1.475–7.563)	0.004
Under-five children				
≤ 5	1		1	
> 5	4.06	< 0.001	4.442 (2.000–9.866)	< 0.001
Complimentary feeding during the first 6 months				
Yes	1		1	
No	2.314	0.007	2.475 (0.8–6.074)	0.48
Dietary Diversity				
Diverse	1		1	
Not diverse	1.967	0.013	1.931 (0.906–4.116)	0.088
Child Immunization				
Fully vaccinated	1		1	
Unvaccinated	4.242	< 0.001	3.641(1.618–8.198)	0.002
Pneumonia				
Yes	3.906	< 0.001	5.611(2.488–12.651)	< 0.001
No	1		1	
Diarrhea				
Yes	1		1	
No	5.425	< 0.001	4.680 (2.169–10.097)	< 0.001
Access to sanitation and hygiene				
Yes	1		1	
No	3.085	< 0.001	3.181(1.462–6.934)	0.004
Household food security				
Insecure	3.830	0.015	9.460 (2.095–42.712)	0.003
Secure	1			

## Discussion

We aimed to describe the extent of the SAM outbreak and identify risk factors associated with the current outbreak in the Dubti District of the Afar Region. A total of 442 cases and 39 deaths were reported. The AR was the highest among male patients (320/1,000 population), infants aged 6–11 months (330/1,000 population), and residents of Galimeda (350/1,000 population). The outbreak spanned 23 weeks, and the epidemic curve suggested a continuous common-source type of outbreak. A large family size, a high number of under-five children, a lack of vaccination, a lack of access to sanitation and hygiene, acute illnesses such as pneumonia, diarrheal disease, and household food insecurity were the factors associated with this outbreak.

The reported case fatality rate (CFR) for SAM in the current outbreak was 8.82%. The findings are consistent with CFRs reported in studies conducted in Addis Ababa, Ethiopia (10%) (31); at Felege Hiwot Hospital, Bahr Dar, Ethiopia (11.3%) (32); in Nigeria (8.5%) (33); and at St. Mary’s Hospital, Uganda (12.6%) (34). However, the CRF in this study was higher than the observed CFR in studies conducted at Hiwot Fana Specialized Hospital, Ethiopia (2.1%) (35), and in rural Jharkhand and Odisha, eastern India (1.2%) (36). It also exceeds the WHO’s and Ethiopia’s target for SAM management, which reports a CFR of less than 5% (37, 38). Variations in the demographic and underlying clinical characteristics of children, treatment protocols, resource availability, medical supplies, and quality of care may have contributed to the observed differences in CFR rates across various settings. Furthermore, the findings suggest a need to improve the quality of care as there may be gaps in healthcare providers’ adherence to treatment protocols, training, and resource availability.

The children in families with more than five members had 3.34 times greater odds of experiencing SAM compared to the children in smaller families. Similarly, the odds of SAM were 29.4% higher in households with more than five under-five children. These findings are consistent with studies conducted in the Libo Kemekem district, Amhara region (39), Benna Tsemay district, southern Ethiopia (40), and Bangladesh (41). This may be attributed to increased economic strain and the sharing of limited food among family members in households with larger family sizes and more children, which can lead to poor nutritional status.

The odds of SAM were 5.6 times greater among the children who had pneumonia compared to their counterparts. Pneumonia was a common comorbidity among severely malnourished children in a study conducted in Bangladesh (42). This may be attributed to malnutrition weakening the body’s immune system, reducing physical activity, and increasing susceptibility to pneumonia. In addition, insensible dehydration due to rapid breathing or fever, combined with decreased appetite from pneumonia, may have contributed to SAM.

Lack of access to sanitation and hygiene was associated with a 3.2-fold greater odds of SAM. Similarly, children with diarrheal disease had a 3.6-fold greater chance of experiencing SAM. These findings are consistent with studies conducted in the districts of Dermot, Kalafo, and Enebsie Sarmidr in Ethiopia (43–45), as well as Vadodara, India (46). This may be explained by appetite loss, poor digestion, malabsorption, and electrolyte loss due to diarrheal disease, which can result in acute weight loss and malnutrition.

In the present study, the immunization status of the children was associated with the development of SAM. The non-immunized children had a 4.7-fold greater risk of experiencing SAM. This finding aligns with studies conducted in the Benishangul-Gumz (47) and Somali regional states of Ethiopia (48) and Zambia (49). This may be explained by the fact that non-immunized children are more likely to contract pneumonia and diarrheal disease due to missed vaccinations.

Children from food-insecure households were 9.5 times more likely to develop SAM compared to their counterparts. Several studies support the positive association between household food insecurity and SAM. Specifically, household food insecurity was associated with a fourfold increased risk of SAM in studies conducted in Leqa Dulacha District, Oromia region, Ethiopia, and in two districts (Terai and Jhapa) in Nepal (21, 23). In addition, the likelihood of a child developing SAM was 1.8 times greater among food-insecure households in a study conducted in Mao City, Chad (25). This may be attributed to food-insecure households experiencing food shortages, which lead to insufficient dietary intake for children in terms of both quantity and quality, thereby increasing their risk of severe acute malnutrition.

## Limitations of the study

The study has some limitations. First, potential recall bias might have affected the reporting of past events. However, we mitigated this by using reference calendars, such as holidays, to assist the mothers/caregivers in their recall. Second, owing to the reciprocal causation relationships between SAM and pneumonia as well as between SAM and diarrhea, a child may have acquired diarrheal disease and pneumonia after developing SAM. However, we addressed this by asking the mothers/caregivers about the temporal sequence of these conditions. Third, recumbent length measurements for young children aged 6–23 months may have been influenced by their inability to lie completely straight, thus potentially affecting the reliability of the results. To mitigate this bias, we strictly adhered to standardized anthropometric procedures and involved two individuals in the measurement process to ensure the maximum validity of the measurements and the reliability of the results. Finally, this study did not examine specific missed vaccines associated with SAM or the potential relationship between family income/wealth index and SAM.

### Areas for further research

Future research could delve deeper into the relationship between the family income/wealth index and SAM. In addition, investigating the specific child vaccines that were missed and their potential association with SAM would provide valuable insights for targeted interventions. Finally, future research should aim to conduct longitudinal studies to establish more definitive causal relationships between SAM and pneumonia, as well as SAM and diarrhea. By exploring these areas, researchers can contribute to a more comprehensive understanding of the factors contributing to SAM and develop more effective prevention and treatment strategies.

## Conclusion

The AR was the highest among male patients, infants aged 6–11 months, and residents of Galimeda. The epidemic curve suggested a continuous common-source type of outbreak. In this study, the CFR was higher than the WHO’s and Ethiopia’s targets for SAM management. Risk factors for the current outbreak included households with more than five members, more than five under-five children, lack of vaccination, diarrheal disease, pneumonia, limited access to sanitation and hygiene, and household food insecurity. The findings demonstrate the need for multisectoral and multidisciplinary collaboration to improve essential child health services, access to WASH, and household food security through economic empowerment.

## Data Availability

The original contributions presented in the study are included in the article/Supplementary material, further inquiries can be directed to the corresponding author.
