# Supplementary material for: Investigating a severe acute malnutrition outbreak in Dubti District, Awsiresu Zone, Afar Region, Northeast Ethiopia (2022)
Source: Front Public Health. 2024 Nov 7;12:1475104. doi: 10.3389/fpubh.2024.1475104 (PMC11578949; doi:10.3389/fpubh.2024.1475104)
Supplement: Supplementary file 1 [file Table_1.docx]

**Questionnaire**

**Section one: Identification**

Participant code_________________________

Kebele_________________________________

Village_________________________________

Date of data collection_____________________

Data collector_____________________________ Signature:______________________

Supervisor:________________________________ Signature:______________________
Date_____________________________

**Section two: Line list: descriptive study**

| S. No | Questions | Responses | Skip/remark |
| --- | --- | --- | --- |
| 1 | Age (in months) | _____________________ |  |
| 2 | Sex of child | 1. Male 2. Female |  |
| 3 | Weight for length | ________________ | For 6-23 months |
| 4 | Weight for height |  | > 23 months |
| 5 | Mid upper arm circumference | ________________ |  |
| 6 | Bilateral pitting edema | 1. Yes 2. No |  |
| 7 | Diarrhea | 1. Yes 2. No |  |
| 8 | Pneumonia | A. Yes  B. No |  |
| 9 | Fever | A. Yes  B. No |  |

**Section three: Questionnaire for analytic study**

| **Socio-demographic Characteristics** | | | **Skip** |
| --- | --- | --- | --- |
| S. No | Questions | Responses |  |
| 1 | Respondent status | 1. Case 2. Control |  |
| 2 | Sex of child | 1. Male 2. Female |  |
| 3 | Age of the child (in months) | ____________________ |  |
| 4 | Age of the mother/caregiver | ______________________ |  |
| 5 | Mothers/caregivers marital status | 1. Married 2. Divorced 3. Widowed 4. Others (specify)_________ |  |
| 6 | Mothers/caregivers occupation | 1. Housewife 2. Herding livestock 3. Employed 4. Others (specify)_________ |  |
| 7 | Mothers/caregivers ethnicity | 1. Afar 2. Amhara 3. Tigre 4. Others (specify)____________ |  |
| 8 | Mothers/caregivers religion | 1. Muslim 2. Orthodox 3. Protestant 4. Others (specify)___________ |  |
| 9 | Mothers/caregivers education | 1. No formal education 2. Primary (1-8) 3. Secondary (9-12) 4. College and above |  |
| 10 | Household family size | -------------------- |  |
| 11 | Number of under-five children | ---------------- |  |
| **Anthropometric measurements of child** | | |  |
| 1 | Weight (kg) | ____________ |  |
| 2 | Height (length) in (cm) | _____________ |  |
| 3 | Weight for height or weight for length | ________________ |  |
| 4 | Mid upper arm circumference | ________________ |  |
| 5 | Bilateral pitting edema | 1. Yes 2. No |  |
| **Child feeding practices** | | |  |
| 1 | Birth order | 1. First 2. Second 3. Third 4. Fourth 5. Fifth 6. Sixth and above |  |
| 2 | Initiation of breast feeding | 1. Within one hour 2. Hours later |  |
| 3 | Pre lacteal feeding | A. Yes  B. No |  |
| 4 | If yes to Q. | 1. Animal milk 2. Butter 3. Water 4. Others (specify)________ |  |
| 5 | Colostrum feeding | 1. Yes 2. No |  |
| 6 | Exclusive breast feeding during the first six months of life | 1. Yes 2. No |  |
| 7 | Currently breast feeding | 1. Yes 2. No |  |
| 8 | Initiation of complementary feeds at six month | A. Yes  B. No |  |
| **Child health-related characteristics** | | |  |
| 1 | Child immunization | 1. Fully vaccinated 2. Partially/unvaccinated |  |
| 2 | Is child has cough? | 1. Yes 2. No | If no, skip to Q5 |
| 3 | If yes to Q2, measure respiratory rate | ___________breaths/minute |  |
| 5 | Is child has had fever? | 1. Yes 2. No |  |
| 6 | Is child has diarrhea? | 1. Yes 2. No |  |
| **Household access to water supply, sanitation and hygiene** | | |  |
| S/N | Questions | Responses |  |
| 1 | Household access to safe drinking water | 1. Yes 2. No | If no, skip to Q3 |
| 2 | If yes to Q1, source of drinking water? | 1. Protected well 2. Un protected |  |
| 3 | Presence of latrine | 1. Yes 2. No |  |
| 4 | Household access to materials for sanitation and hygiene including soap and hand washing utilities? | 1. Yes 2. No |  |
| **Dietary diversity** | | |  |
| S/N | Questions | Responses | Skip |
|  | The last 24 hours child’s feeds consists of: |  |  |
| 1 | Grains, roots and tubers | 1. Yes 2. No |  |
| 2 | Legumes and nuts | 1. Yes 2. No |  |
| 3 | Dairy products (milk, yogurt, cheese) | 1. Yes 2. No |  |
| 4 | Flesh foods (meat, fish, poultry and liver/organ meats) | 1. Yes 2. No |  |
| 5 | Eggs | 1. Yes 2. No |  |
| 6 | Vitamin-A rich fruits and vegetables | 1. Yes 2. No |  |
| 7 | Other fruits and vegetables | 1. Yes 2. No |  |
|  | Dietary Diversity Score | ________________________ | |
| **Household Food Insecurity Access Scale (HFIAS) Measurement Tool** | | | |

| No | Question | Response Options | Code |
| --- | --- | --- | --- |
| 1. | In the past four weeks, did you worry that your household would not have enough food? | 0= No (skip to Q2)  1=Yes |  |
| 1.a | How often did this happen? | 1= Rarely (once or twice in the past four weeks)  2= Sometimes (three to ten times in the past four weeks  3= Often (more than ten times in the past four weeks) |  |
|  | In the past four weeks, were you or any household member not able to eat the kinds of foods you preferred because of a lack of resources? | 0= No (skip to Q3)  1=Yes |  |
| 2.a | How often did this happen? | 1= Rarely (once or twice in the past four weeks)  2= Sometimes (three to ten times in the past four weeks)  3= Often (more than ten times in the past four weeks) |  |
| 3. | In the past four weeks, did you or any household member have to eat a limited variety of foods due to a lack of resources? | 0= No (skip to Q4)  1=Yes |  |
| 3.a | How often did this happen? | 1= Rarely (once or twice in the past four weeks)  2= Sometimes (three to ten times in the past four weeks)  3= Often (more than ten times in the past four weeks) |  |
| 4. | In the past four weeks, did you or any household member have to eat some foods that you really did not want to eat because of a lack of resources to obtain other types of food? | 0= No (skip to Q5)  1=Yes |  |
| 4.a | How often did this happen? | 1= Rarely (once or twice in the past four weeks)  2= Sometimes (three to ten times in the past four weeks)  3= Often (more than ten times in the past four weeks) |  |
| 5. | In the past four weeks, did you or any household member have to eat a smaller meal than you felt you needed because there was not enough food? | 0= No (skip to Q6)  1=Yes |  |
| 5.a | How often did this happen? | 1= Rarely (once or twice in the past four weeks)  2= Sometimes (three to ten times in the past four weeks)  3= Often (more than ten times in the past four weeks) |  |
| 6. | In the past four weeks, did you or any other household member have to eat fewer meals in a day because there was not enough food? | 0= No (skip to Q7)  1=Yes |  |
| 6.a | How often did this happen? | 1= Rarely (once or twice in the past four weeks)  2= Sometimes (three to ten times in the past four weeks)  3= Often (more than ten times in the past four weeks) |  |
| 7. | In the past four weeks, was there ever no food to eat of any kind in your household because of lack of resources to get food? | 0= No (skip to Q8)  1=Yes |  |
|  |  | 1= Rarely (once or twice in the past four weeks)  2= Sometimes (three to ten times in the past four weeks)  3= Often (more than ten times in the past four weeks) |  |
| 7.a | How often did this happen? |  |  |
| 8. | In the past four weeks, did you or any household member go to sleep at night hungry because there was not enough food? | 0= No (skip to Q9)  1=Yes |  |
| 8.a | How often did this happen? | 1= Rarely (once or twice in the past four weeks)  2= Sometimes (three to ten times in the past four weeks)  3= Often (more than ten times in the past four weeks) |  |
| 9. | In the past four weeks, did you or any household member go a whole day and night without eating anything because there was not enough food? | 0= No (questionnaire is finished)  1=Yes |  |
| 9.a | How often did this happen? | 1= Rarely (once or twice in the past four weeks)  2= Sometimes (three to ten times in the past four weeks)  3= Often (more than ten times in the past four weeks) |  |
| HFIA category | 1. category one: food secure | if [(Q1a=0 or Q1a=1) and Q2=0 and Q3=0 and Q4=0 and Q5=0 and Q6=0 and Q7=0 and Q8=0 and Q9=0] | |
|  | 1. Category two: Mildly Food Insecure Access | if [(Q1a=2 or Q1a=3 or Q2a=1 or Q2a=2 or Q2a=3 or Q3a=1 or Q4a=1)  and Q5=0 and Q6=0 and Q7=0 and Q8=0 and Q9=0] | |
|  | 1. Category three: Moderately Food Insecure Access | if [(Q3a=2 or Q3a=3 or Q4a=2 or Q4a=3 or Q5a=1 or Q5a=2 or Q6a=1 or  Q6a=2) and Q7=0 and Q8=0 and Q9=0] | |
|  | 1. Category four: Severely Food Insecure Access | if [Q5a=3 or Q6a=3 or Q7a=1 or Q7a=2 or Q7a=3 or Q8a=1 or Q8a=2 or  Q8a=3 or Q9a=1 or Q9a=2 or Q9a=3] | |
